# Biogenic synthesis and characterization of lanthanum oxide nanoparticles using citrus sinensis peel waste for antifungal applications in crop protection

**DOI:** 10.3389/ffunb.2026.1925291

**Published:** 2026-09-11

**Authors:** Shakthika Ramesh, Appireddy Khyathi, Hema sree Mandapati, Vikram S., Varshini Kumar, Theivasigamani Parthasarathi

**Affiliations:** Department of Genetics and Plant Breeding, Vellore Institute of Technology (VIT) School of Agricultural Innovations and Advanced Learning (VAIAL), Vellore Institute of Technology, Vellore, Tamil Nadu, India

**Keywords:** biocontrol, green synthesis, La_2_O_3_ nanoparticles, orange peel extract, phytopathogenic fungi

## Abstract

**Introduction:**

Green synthesis of metal oxide nanoparticles using agricultural waste offers a sustainable approach to developing alternative antifungal agents. This study investigated the potential of lanthanum oxide (La_2_O_3_) nanoparticles synthesized using *Citrus sinensis* peel extract as an eco-friendly antifungal agent against important phytopathogens.

**Methods:**

La_2_O_3_ nanoparticles were synthesized using *C. sinensis* peel extract and characterized using X-ray diffraction (XRD), Fourier-transform infrared spectroscopy (FTIR), UV–visible spectroscopy (UV–Vis), field-emission scanning electron microscopy (FESEM), and energy-dispersive X-ray spectroscopy (EDX). Their antifungal activity was evaluated against *Alternaria solani* and *Colletotrichum capsici* at different concentrations and compared with the commercial fungicide SAAF.

**Results:**

The synthesized La_2_O_3_ nanoparticles exhibited a crystalline cubic structure with characteristic physicochemical properties. FESEM analysis revealed irregular, flake-like to quasi-spherical particles, while EDX confirmed the presence of lanthanum, oxygen, and carbon. The nanoparticles demonstrated concentration-dependent antifungal activity against both pathogens. *A. solani* was more susceptible, with complete growth inhibition at 5 mg/mL, whereas inhibition of C. capsici reached 86.8% at 15 mg/mL. Complete suppression of sporulation was observed in La_2_O_3_-treated cultures. Against *A. solani*, La_2_O_3_ nanoparticles at 15 mg/mL, earlier than the commercial fungicide at 5 mg/mL.

**Discussion:**

The findings demonstrate that C. sinensis peel-derived La2O3 nanoparticles possess substantial antifungal activity, particularly against *A. solani*. The combined physicochemical characterization and antifungal results support their potential as an eco-friendly biocontrol agent while highlighting the need for further optimization and evaluation against comparatively more resistant pathogens such as *C. capsici*.

## 1 Introduction

Nanotechnology represents one of the most transformative scientific disciplines of the 21^st^ century, enabling the creation and manipulation of matter at the atomic and molecular scale (1–100 nm) to develop novel materials with unprecedented properties (Iravani et al., 2014; Mittal et al., 2013). Nanoparticles exhibit optical, electrical, catalytic, and antimicrobial properties that differ substantially from their bulk counterparts, largely due to their high surface-area-to-volume ratio and quantum-scale effects (Slavin and Bach, 2022).

A range of nanomaterial classes have been explored as antimicrobial and antifungal agents in agriculture, each with distinct advantages and limitations. Noble metal nanoparticles, particularly silver (AgNPs) and copper (CuNPs), have been the most extensively studied, owing to their potent broad-spectrum antimicrobial activity via ion release and reactive oxygen species (ROS) generation (Ahmed et al., 2016; Slavin and Bach, 2022). However, their agricultural application is constrained by high production cost, documented phytotoxicity at elevated concentrations, and concerns over bioaccumulation and non-target toxicity to soil microbiota and aquatic organisms (Dikshit et al., 2021). Transition metal oxide nanoparticles, such as zinc oxide (ZnO) and copper oxide (CuO), offer lower cost and comparable antimicrobial efficacy through ROS-mediated mechanisms, but similarly raise concerns regarding dose-dependent phytotoxicity and soil accumulation with repeated agricultural use (Mittal et al., 2013). Titanium dioxide (TiO_2_) nanoparticles exhibit strong UV-activated photocatalytic antimicrobial activity, though this photodependence limits efficacy under field conditions with variable light exposure (de López et al., 2021; Shang et al., 2022). Iron oxide nanoparticles (Fe_3_O_4_) offer favorable biocompatibility and magnetic recoverability, but generally show comparatively weaker intrinsic antifungal activity relative to other metal oxides (Afrouz et al., 2023; Serov et al., 2024).

Rare-earth metal oxides constitute a comparatively underexplored nanomaterial class for agricultural antimicrobial use. Among these, lanthanum oxide (La_2_O_3_) has attracted growing interest owing to its favorable combination of properties: high chemical stability, a wide bandgap suited to sustained oxidative activity, and applications spanning catalysis, biotechnology, environmental remediation, and agriculture (Mittal et al., 2013; Singh et al., 2016). Critically, lanthanum-based compounds have generally demonstrated a more favorable toxicological profile than silver or copper-based nanoparticles in comparative studies, with lower reported cytotoxicity and reduced tendency for bioaccumulation at equivalent antimicrobial concentrations (Kishore et al., 2025a), although rare-earth element toxicity in soil and aquatic systems remains an active area of investigation and has not been fully characterized for green-synthesized formulations (Ifijen et al., 2025).

This comparatively favorable safety profile, combined with La_2_O_3_’s established but underexploited biological activity, motivated its selection over more extensively studied but higher-toxicity-risk nanomaterials such as AgNPs and CuNPs for the present study (Jadhav et al., 2025). Nonetheless, as with all metal oxide nanomaterials, the environmental fate and long-term ecotoxicological impact of La_2_O_3_ nanoparticles at agricultural application scales require dedicated future investigation before large-scale field deployment (Adeel et al., 2021). Despite these favorable properties, the direct antifungal activity of La_2_O_3_ nanoparticles remains comparatively underexplored relative to other metal oxide nanomaterials (Ameen et al., 2021). Existing literature has primarily focused on La_2_O_3_’s utility in plant growth promotion, stress mitigation, and nutrient uptake enhancement (Gao et al., 2025), while its potential as a direct biocontrol agent against fungal phytopathogens has received limited systematic investigation. The few available reports on La_2_O_3_ antifungal or antimicrobial activity have largely relied on chemically synthesized nanoparticles, and rarely include head-to-head efficacy comparisons against agriculturally relevant pathogens or established commercial fungicides, leaving an important gap regarding its practical viability as a biocontrol agent.

Agricultural productivity is increasingly threatened by fungal phytopathogens, which inflict enormous economic losses globally, estimated at over $220 billion annually. *Alternaria solani* and *Colletotrichum capsici* are particularly devastating pathogens causing early blight in tomatoes and anthracnose in chili peppers, respectively, resulting in yield reductions of 20–90% under favorable disease conditions (Than et al., 2008). Traditional disease management relies heavily on synthetic fungicides, which pose substantial environmental and public health concerns, including pesticide resistance development (Fisher et al., 2018), non-target toxicity, soil microbiota disruption, and bioaccumulation in food chains (Dikshit et al., 2021). Even when effective against target pathogens, such as in trials evaluating chemical fungicides against Botrytis cinerea-induced grey mold (Jatoi et al., 2022), synthetic fungicides commonly leave residues that persist beyond harvest; these residues, particularly when combined with chemical food preservatives, raise ongoing food safety concerns for fresh produce (Aguilar-Marcelino et al., 2023). Consequently, there is an urgent need to develop alternative disease management strategies that are environmentally benign, economically viable, and effective against resistant pathogen strains (Fisher et al., 2018).

Green nanotechnology, also termed “biological” or “eco-friendly” nanotechnology, offers a paradigm shift by employing renewable biological resources and non-toxic reducing agents for nanoparticle synthesis, in contrast to the chemical reduction routes used for many of the nanomaterials discussed above (Mittal et al., 2013; Singh et al., 2016). Plant-based extracts represent a particularly promising avenue, as they contain diverse secondary metabolites, including polyphenols, flavonoids, alkaloids, and ascorbic acid, that serve as natural reducing and stabilizing agents (Pandey and Rizvi, 2009; Dai and Mumper, 2010; Scalbert et al., 2005). This approach eliminates hazardous chemical reducing agents (such as sodium borohydride and hydrazine) and high-temperature processing requirements associated with conventional chemical synthesis (Ahmed et al., 2016), thereby reducing the environmental footprint and operational costs while improving worker safety (Dikshit et al., 2021).

*Citrus sinensis* (orange) represents an abundant agricultural biomass resource, with global production exceeding 50 million metric tons annually. Citrus sinensis (sweet orange) represents an abundant agricultural biomass resource, with global orange production exceeding 50 million metric tons annually. Orange processing generates substantial quantities of peel-rich waste, which is often discarded or underutilized despite its potential as a valuable source of bioactive compounds (Gavahian et al., 2019) rich phytochemical composition (Pandey and Rizvi, 2009). Recent studies have demonstrated the utility of citrus peel extracts in synthesizing metal nanoparticles for various applications (Hanif et al., 2024; Rashmi et al., 2022); however, comprehensive investigations combining detailed characterization with systematic evaluation of antifungal efficacy against agriculturally significant pathogens remain limited (Hanif et al., 2024; Rashmi et al., 2022; Perumal et al., 2025). Citrus peel extracts themselves, independent of nanoparticle formulation, have also shown direct antifungal bioactivity: *C. limon* and *C. sinensis* peel extracts were recently reported to suppress potato rot-causing fungal pathogens through their phytochemical content (Hajji-Hedfi et al., 2025), supporting the rationale that *C. sinensis* derived phytoconstituents used in the present study may contribute directly to antifungal activity independent of, or in addition to, the nanoparticle’s inorganic component.

To our knowledge, this is the first study to synthesizeLa_2_O_3_ nanoparticles specifically from *C. sinensis* peel extract and to evaluate their antifungal efficacy against *A. solani* and *C. capsici*, two economically important phytopathogens not previously tested against La_2_O_3_ nanoparticles in the existing literature. This work is further distinguished by its integration of comprehensive physicochemical characterization (XRD, FTIR, UV-Vis, FESEM, EDX) with direct, concentration- and time-resolved antifungal efficacy testing benchmarked against a commercial fungicide standard, an evaluative framework rarely applied together in prior green-synthesis La_2_O_3_ studies. In addition, the combined FTIR and EDX evidence of retained surface phytochemicals supports a bio-hybrid nanostructure model in which the antifungal mechanism may derive from both the inorganic oxide core and the retained organic phytoconstituents, a dual-mechanism perspective not explicitly addressed in comparable prior work. By valorizing an abundant agricultural waste stream into a functional, comparatively lower-toxicity-risk antifungal nanomaterial, this study contributes both a practical waste-to-value pathway and new pathogen-specific efficacy data to the emerging field of rare-earth-oxide-based green nanotechnology in agriculture.

The specific objectives of this study were to: (i) optimize a green synthesis protocol for La_2_O_3_ nanoparticles using *C. sinensis* peel extract; (ii) characterize the synthesized nanoparticles through complementary analytical techniques (XRD, FTIR, UV-Vis, FESEM, and EDX); (iii) evaluate their antifungal efficacy against A. solani and C. capsici across a range of concentrations; and (iv) compare this efficacy with a commercial fungicide to assess their practical potential as a biocontrol agent.

## 2 Materials and methods

### 2.1 Preparation of green synthesis of lanthanum oxide nanoparticles

Orange (*Citrus sinensis*) peel waste was collected from the campus canteen of Vellore Institute of Technology (VIT), Vellore, Tamil Nadu, India, as a discarded by-product of fruit consumption. The fruit was identified as the commercial sweet orange variety based on standard morphological characteristics; as *C. sinensis* is a widely cultivated commercial species, formal herbarium voucher deposition was not required. Lanthanum nitrate La(NO_3_)_3_ was procured from SRL (Sisco Research Laboratories Pvt. Ltd., India). Whatman Grade 1 filter paper (Whatman) was used for filtration during extract preparation. Potato dextrose agar (PDA) was procured (HiMedia Laboratories Pvt. Ltd., India). Pure cultures of *Alternaria solani* and *Colletotrichum capsici* were obtained from the Plant Pathology Laboratory, Vellore Institute of Technology (VIT), Vellore, Tamil Nadu, India. The commercial fungicide SAAF (Carbendazim 12% + Mancozeb 63% WP) (UPL Limited, India) and used as the positive control.

**Figure 1:**
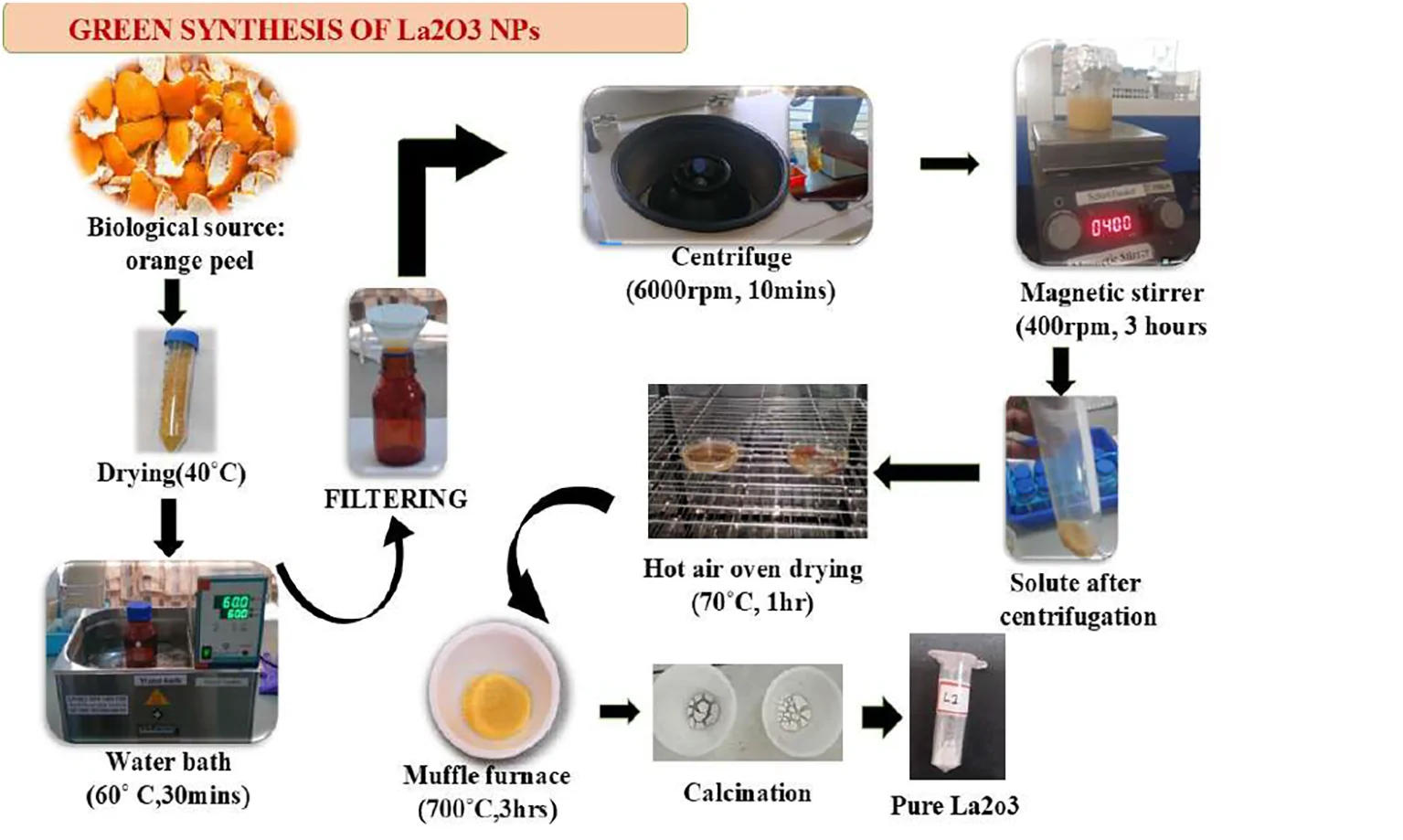
Schematic representation of the green synthesis of La_2_O_3_ nanoparticles using *Citrus sinensis* peel extract.

Freshly collected peels were chopped into small pieces and dried in a hot air oven at 40 °C and ground into powder form. For extract preparation, grinded orange peel powder was added to distilled water and then placed in water bath at 60°Cfor 15 mins. The prepared extract was filtered with Whatman No. 1 filter paper and then the filtered solution was centrifuged at 10,000rpm for 10 mins. 8.66 g of 0.1M Lanthanum Nitrate (La(NO_3_)_3_ was dissolved in 200ml of distilled water. 20ml of Lanthanum Nitrate and Plant extract was added and a makeup volume of 40ml was then stirred using a magnetic stirrer for 3hrs at 400rpm at 50 °C, and the pH was brought to 10 by adding 1M NaOH. The solution was centrifuged at 10,000 rpm for about 10 min. The pellet was washed with ethanol to eliminate impurities. After washing, the pellet was spread in a petri plate and then placed in a hot air oven at 70 °C for 1 hour. The dried nanoparticles in the petri dish were scraped off using a spatula and ground in mortar and pestle, then placed in a Muffle Furnace at 700 °C for 3 hours. Obtained nanoparticles are stored in an airtight jar at room temperature (Hanif et al., 2024). The overall procedure for the green synthesis of La₂O₃ nanoparticles using Citrus sinensis peel extract is illustrated in **Figure 1**.

### 2.2 Characterization

#### 2.2.1 UV–visible spectroscopy

The optical properties of the green-synthesized La_2_O_3_ nanoparticles were analyzed using a UV–Visible spectrophotometer (Shimadzu UV-1780, Shimadzu, Japan). The suspension was transferred to a 1-cm path-length quartz cuvette and scanned over the wavelength range of 290–400 nm (Hanif et al., 2024).

#### 2.2.2 FTIR spectroscopy

The functional groups associated with the synthesized La_2_O_3_ nanoparticles and orange peel extract were characterized using an FTIR spectrometer (IRAffinity-1, Shimadzu, Japan).

Spectra were recorded over the range of 400–4000 cm^–1^.The resulting spectra were baseline corrected and analyzed to identify characteristic bands associated with La–O vibrations and phytochemical functional groups potentially involved in nanoparticle synthesis and surface stabilization (Hanif et al., 2024).

#### 2.2.3 X-ray diffraction analysis

The crystalline structure and phase composition of the synthesized La_2_O_3_ nanoparticles were determined using powder X-ray diffraction (XRD; Bruker D8 Advance, Bruker, Germany).

The analysis was performed at room temperature. The obtained diffraction peaks were indexed and compared with standard International Centre for Diffraction Data (ICDD/JCPDS) reference patterns to determine the crystalline phase and phase purity of the synthesized La_2_O_3_ nanoparticles (Hanif et al., 2024).

#### 2.2.4 FESEM analysis

The surface morphology and particle size characteristics of the green-synthesized La_2_O_3_ nanoparticles were examined using field-emission scanning electron microscopy (FESEM; FEI Quanta 250 FEG, Thermo Fisher Scientific, USA). The sample was coated with a thin conductive layer of gold using a sputter coater FESEM imaging was performed at an accelerating voltage of 20 kV, at magnifications of 5,000×, 6,000×, 25,000×, 50,000×, and 100,000× (Hanif et al., 2024 and Maheshwaran et al., 2021).

#### 2.2.5 EDX spectroscopy

The elemental composition of the synthesized La_2_O_3_ nanoparticles was determined using energy-dispersive X-ray spectroscopy (EDX) coupled to the FESEM instrument. EDX spectra were collected from representative nanoparticle regions under the same accelerating voltage of 20 kV. EDX spectra were collected from the sample to confirm the presence of lanthanum (La) and oxygen (O) and to assess the presence of any additional elements originating from the plant extract, substrate, or sample preparation (Maheshwaran et al., 2021).

### 2.3 Preparation of fungal cultures and inoculum standardization

Pure cultures of *Alternaria solani* and *Colletotrichum capsici* were obtained from the Plant Pathology Laboratory, Vellore Institute of Technology (VIT), Vellore, Tamil Nadu, India. The cultures were maintained on potato dextrose agar (PDA; HiMedia Laboratories Pvt. Ltd., India) slants by periodic sub-culturing to maintain culture viability. These isolates are maintained as reference cultures for teaching and research purposes at the source laboratory; as they were obtained from an in-house laboratory collection rather than a national culture repository, they do not carry a deposited accession number. Prior to the antifungal assay, each fungal culture was transferred aseptically onto fresh PDA plates and incubated at 25 ± 2 °C in the dark for 7 days to obtain actively growing and uniform mycelial cultures. The inoculum was standardized based on culture age and mycelial disc size. Briefly, a 5-mm-diameter mycelial disc was excised aseptically from the actively growing margin of each 7-day-old culture using a sterile cork borer. Discs of identical diameter and from comparable actively growing regions were used for all treatments and replicates to ensure a uniform initial inoculum (Singh et al., 2023).

### 2.4 Antifungal activity assay

*Alternaria solani* and *Colletotrichum capsici* were selected as representative agriculturally important phytopathogens for evaluating the antifungal potential of the synthesized La_2_O_3_ nanoparticles. *A. solani* is a major causal agent of early blight in tomato, whereas *C. capsici* is an important causal agent of anthracnose disease in chili, both of which can cause substantial yield and quality losses under favorable environmental conditions (Than et al., 2008). These pathogens were selected to provide a focused evaluation against two economically relevant fungal diseases affecting different vegetable crops and to assess whether nanoparticle efficacy was consistent across distinct phytopathogenic fungi. The present study was therefore designed as a focused proof-of-concept evaluation rather than a comprehensive screening of the antifungal spectrum of La_2_O_3_ nanoparticles. Evaluation against a broader collection of phytopathogenic fungi representing different taxonomic groups and host crops will be necessary in future studies to establish the generality and spectrum of the observed antifungal activity.

The antifungal activity of the biosynthesized La_2_O_3_ nanoparticles was evaluated against *A. solani* and *C. capsici* using the poisoned food technique. La_2_O_3_ nanoparticle suspensions were prepared in sterile deionized water at concentrations of 1, 5, and 15 mg/mL and incorporated into molten PDA medium prior to pouring the plates. The commercial fungicide SAAF (Carbendazim 12% + Mancozeb 63% WP; UPL Limited, India) was incorporated at 5 mg/mL and used as the positive control, whereas PDA without nanoparticles or fungicide served as the untreated control. After solidification of the medium, a 5-mm-diameter mycelial disc from the actively growing margin of a 7-day-old culture was placed at the center of each plate. Three independent replicates were maintained for each treatment. The plates were incubated at 25 ± 2 °C in the dark, and radial colony diameter was measured at 24-h intervals for 7 days.

The percentage inhibition of mycelial growth was calculated using the following equation:

% Inhibition = [(Dc − Dt)/Dc] × 100.

where (Dc) is the colony diameter of the untreated control and (Dt) is the colony diameter of the treatment (Zhu et al., 2026).

### 2.5 Statistical analysis

The results were expressed as mean ± standard deviation (SD) of (n=3). Data were subjected to a one-way analysis of variance (ANOVA) to compare treatment means, and differences between treatments were considered statistically significant at (*p* < 0.001). All statistical analyses and data visualizations were performed using JMP Pro (Version 18).

## 3 Results

The synthesis of La_2_O_3_ nanoparticles from orange peel extract proceeded through a series of distinct colorimetric transformations. Combination of the colorless lanthanum nitrate solution with the dark yellow orange peel extract produced sequential color changes during the 3-hour magnetic stirring step at 50 °C, pH 10. The solution changed from dark yellow to pale yellow within the initial 2–3 hours of stirring. Centrifugation of this solution yielded a tan-colored pellet. After drying (70 °C, 1 hour), the pellet changed from brownish to bright yellow. Following calcination (700 °C, 3 hours), the yellow intermediate converted to a white powder, the final product.

### 3.1 X-ray diffraction

**Figure 2:**
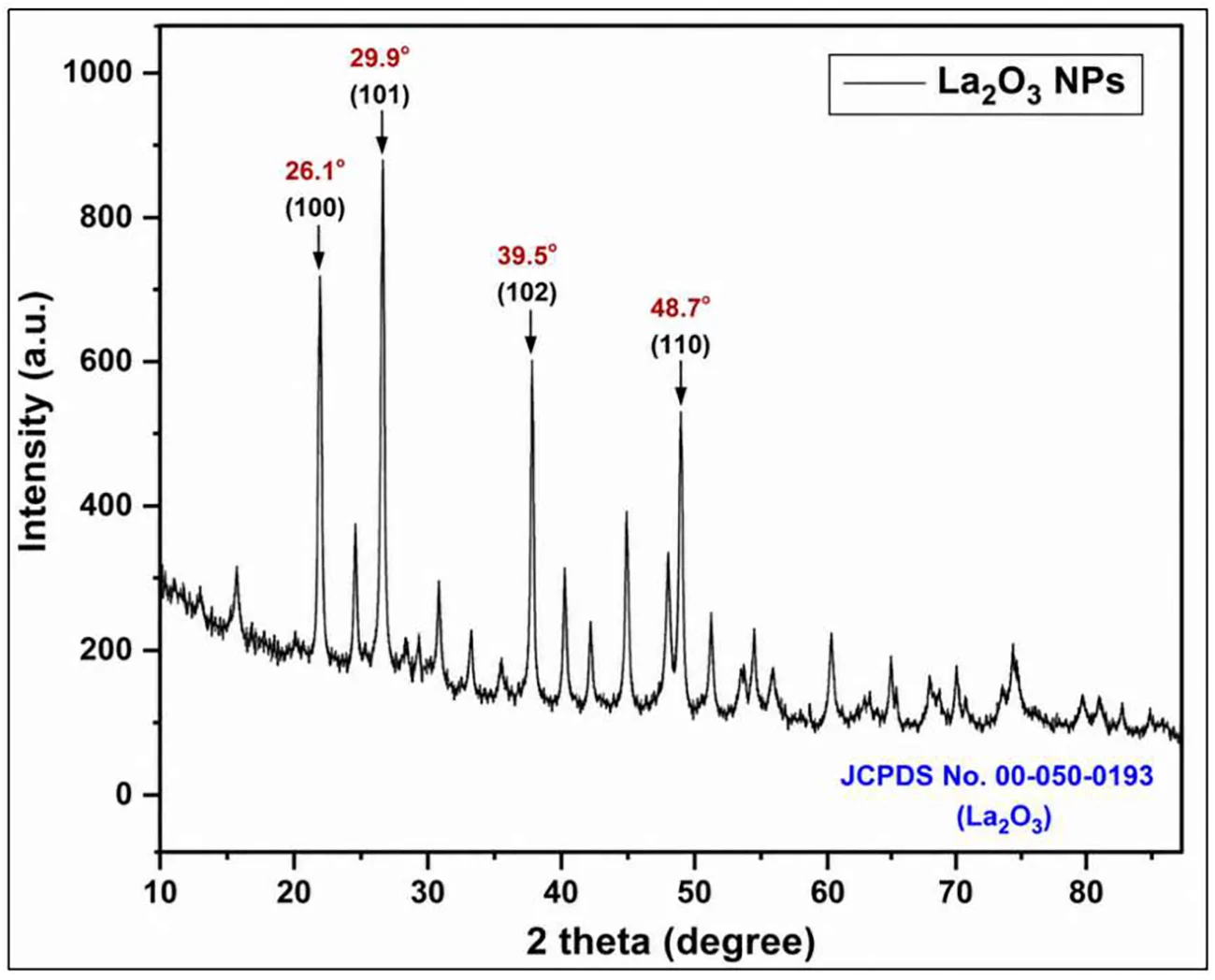
X-ray diffraction (XRD) pattern showing the crystalline nature and phase purity of the synthesized La_2_O_3_ nanoparticles.

The XRD pattern of the synthesized nanoparticles (**Figure 2**) displayed sharp, well-defined Bragg peaks at 2θ values of 26.1°, 29.9°, 39.5°, and 48.7°, corresponding to the (100), (101), (102), and (110) reflection planes of cubic La_2_O_3_. These peak positions matched the ICDD reference standard (JCPDS No. 00-050-0193) for La_2_O_3_.

### 3.2 Fourier-transform infrared spectroscopy

FTIR analysis was performed to identify the functional groups present in the orange peel extract and to confirm their involvement in, or retention following, the biosynthesis of La_2_O_3_ nanoparticles.

**Figure 3:**
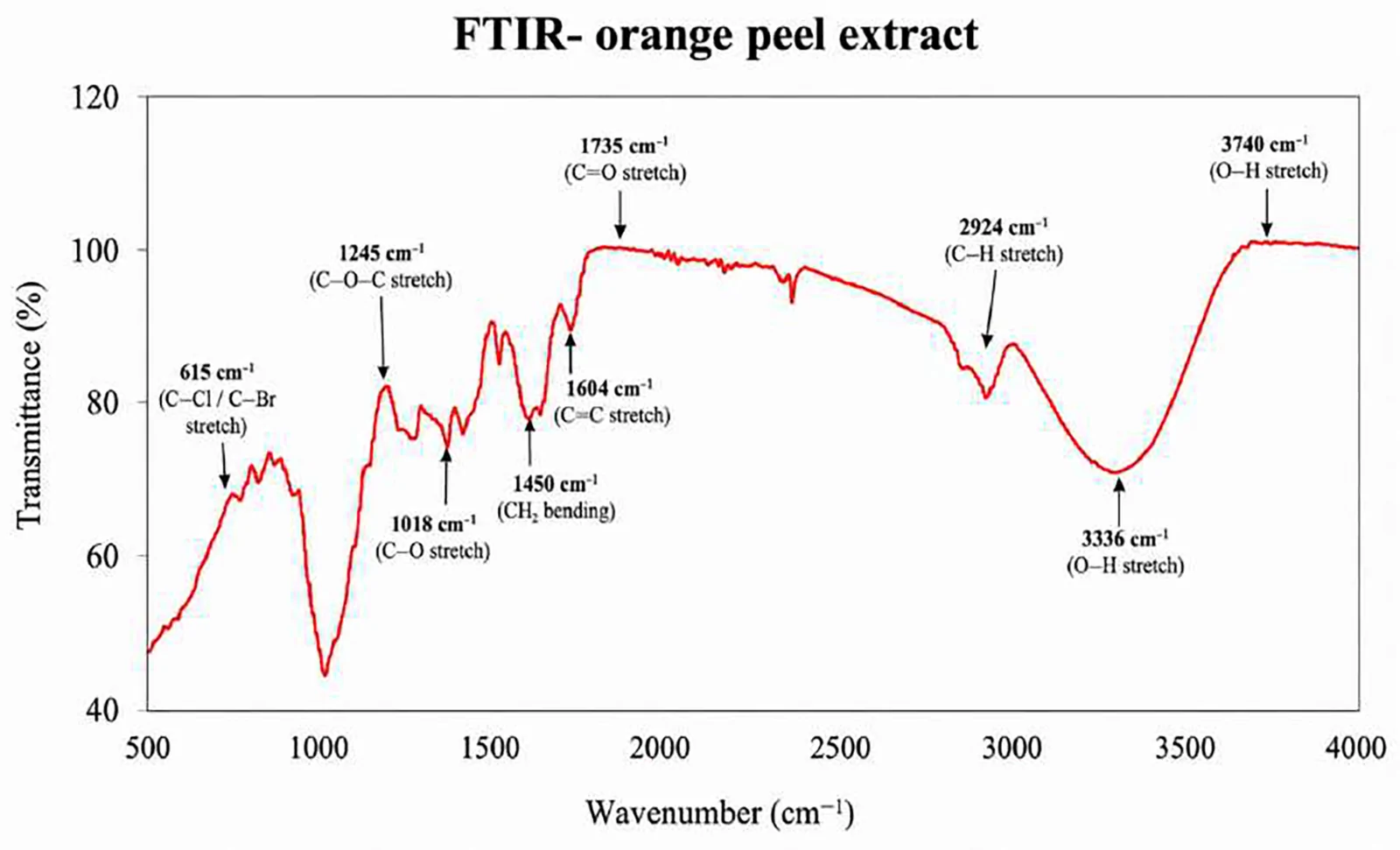
FTIR graph for orange peel extract.

The FTIR spectrum of the orange peel extract (**Figure 3**) displayed a series of absorption bands corresponding to the phytochemical constituents of the extract. Two bands were observed in the O-H stretching region, at 3740 cm^-1^ and 3336 cm^-1^, indicating the presence of free and hydrogen-bonded hydroxyl groups from phenolic and polysaccharide constituents. A band at 2924 cm^-1^was assigned to C-H stretching vibrations of aliphatic groups. A distinct band at 1735 cm^-1^was attributed to C=O stretching, consistent with carbonyl-containing compounds such as esters or carboxylic acids present in the extract. The band at 1604 cm^-1^corresponded to C=C stretching of aromatic rings, characteristic of flavonoid and polyphenolic structures, while the band at 1450 cm^-1^ was assigned to CH_2_ bending vibrations. Bands at 1245 cm^-1^ and 1018 cm^-1^ were attributed to C-O-C and C-O stretching vibrations, respectively, indicative of ether and alcohol linkages in sugars and phenolic compounds. A lower-frequency band at 615 cm^-1^ was assigned to C-Cl/C-Br stretching vibrations, likely arising from trace halogenated compounds in the extract.

**Figure 4:**
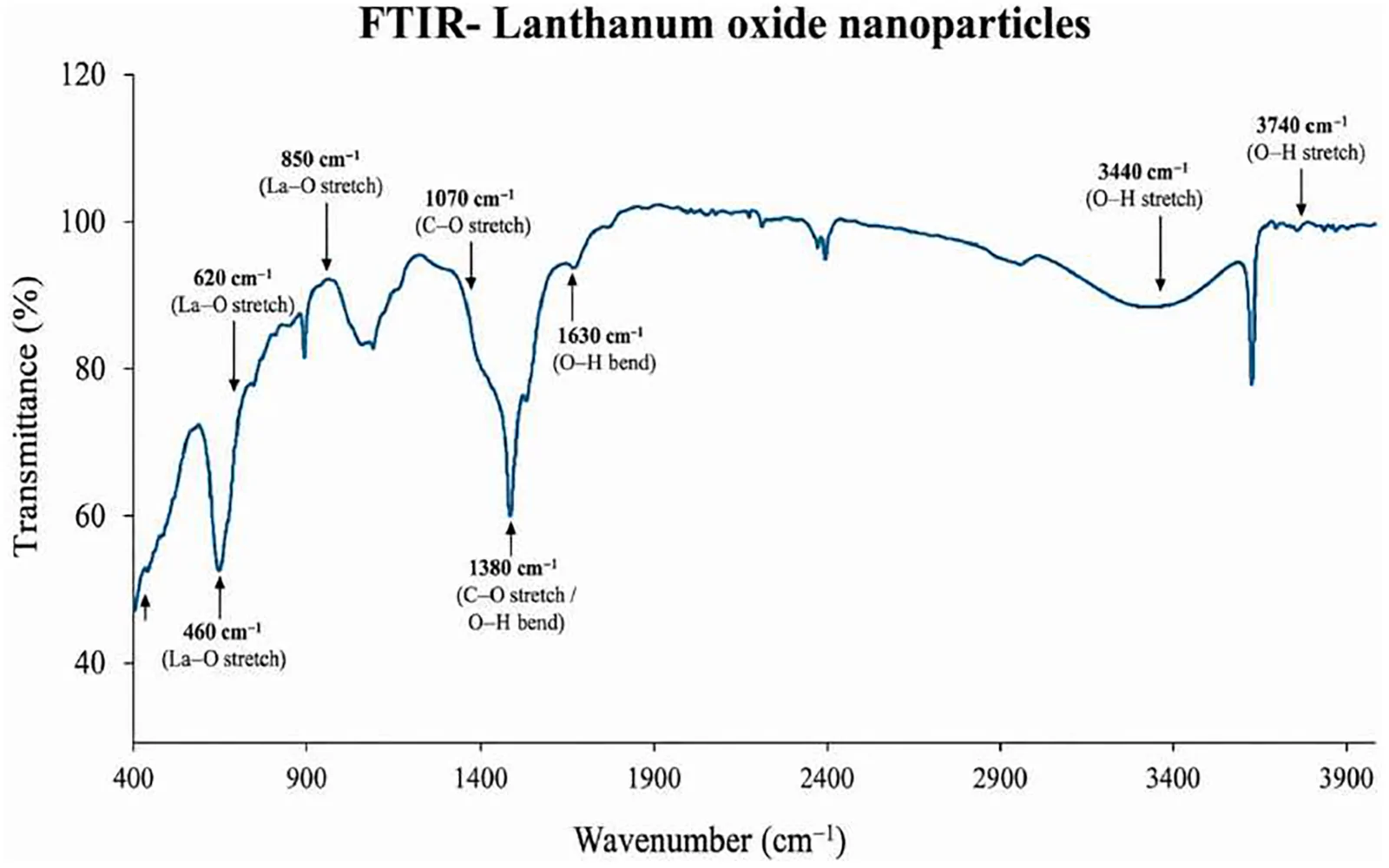
FTIR graph for Lanthanum oxide nanoparticles.

The FTIR spectrum of the synthesized La_2_O_3_ nanoparticles (**Figure 4**) showed a markedly simplified profile relative to the extract, consistent with the thermal decomposition of most organic constituents during calcination. Two O–H stretching bands persisted at 3740 cm^–1^ and 3440 cm^–1^, along with an O–H bending band at 1630 cm^–1^, indicating retention of surface-bound hydroxyl groups and adsorbed moisture. A band at 1380 cm^–1^, assigned to overlapping C–O stretching and O–H bending vibrations, and a further band at 1070 cm^–1^, corresponding to C–O stretching, suggest that residual organic phytoconstituents remained associated with the nanoparticle surface even after calcination. Most notably, three new absorption bands appeared at 890 cm^–1^, 620 cm^–1^, and 460 cm^–1^, all attributed to La–O stretching vibrations, confirming the formation of the lanthanum oxide lattice.

### 3.3 UV-visible spectroscopy

**Figure 5:**
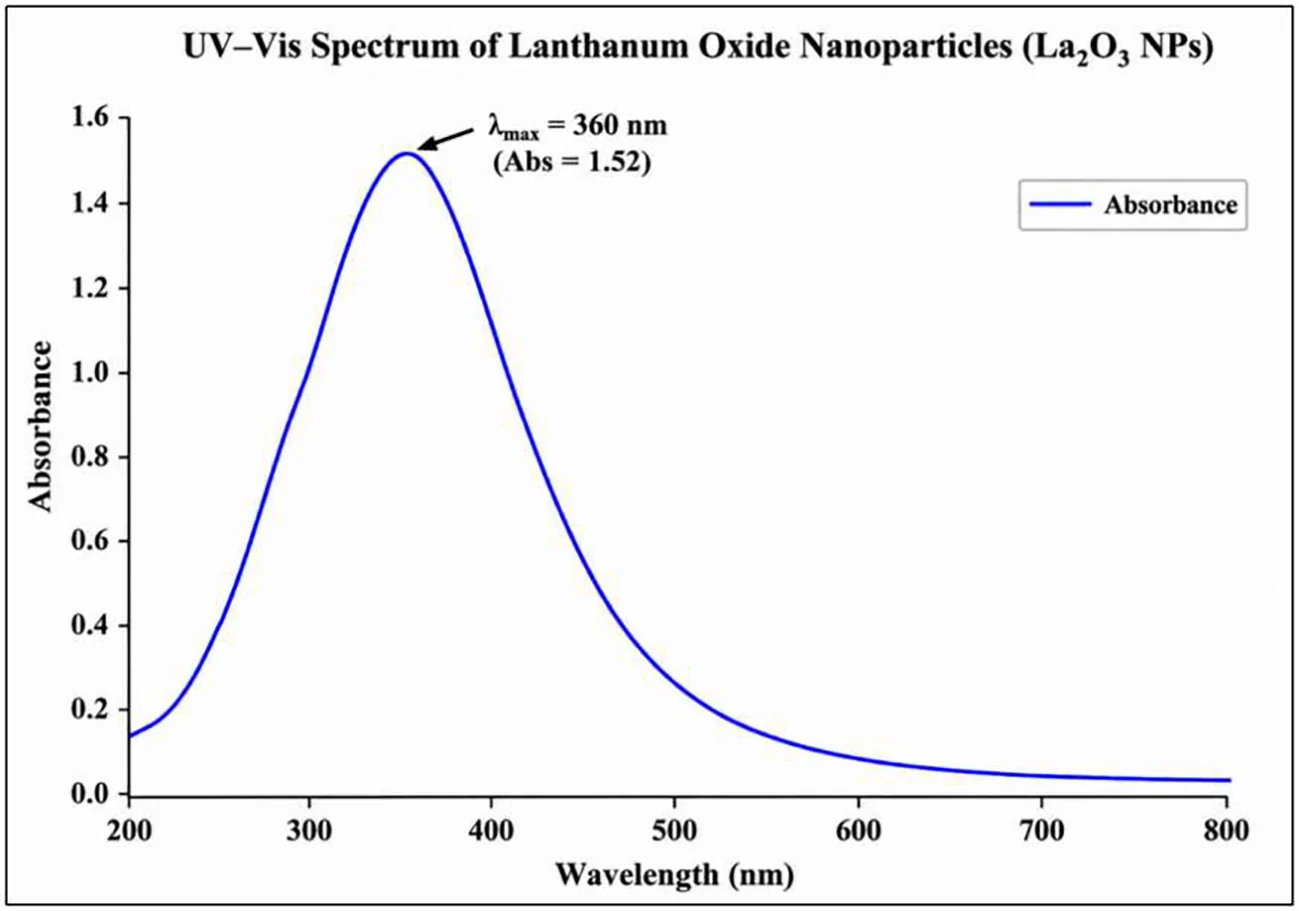
UV-spectrum for lanthanum nanoparticles.

UV-visible spectroscopy was carried out to examine the optical absorption behavior of the synthesized La_2_O_3_ nanoparticles and to assess their electronic structure. The UV-Vis absorption spectrum (**Figure 5**) exhibited a strong, well-defined absorption peak at approximately 360 nm, attributed to the characteristic electronic transitions of the La_2_O_3_ lattice.

Beyond this peak, absorbance declined gradually with increasing wavelength above 400 nm, and only minimal absorbance was recorded across the visible region (400–700 nm). This pattern of strong UV-region absorption coupled with negligible visible-light response is typical of a wide-bandgap metal oxide semiconductor, and indicates that any photocatalytic activity of the nanoparticles would occur predominantly under UV excitation rather than visible light. In addition to the main absorption peak, a broad absorption shoulder was observed in the 290–320 nm region, appearing as a distinct feature separate from the primary 360 nm band.

### 3.4 Field-emission scanning electron microscopy

**Figure 6:**
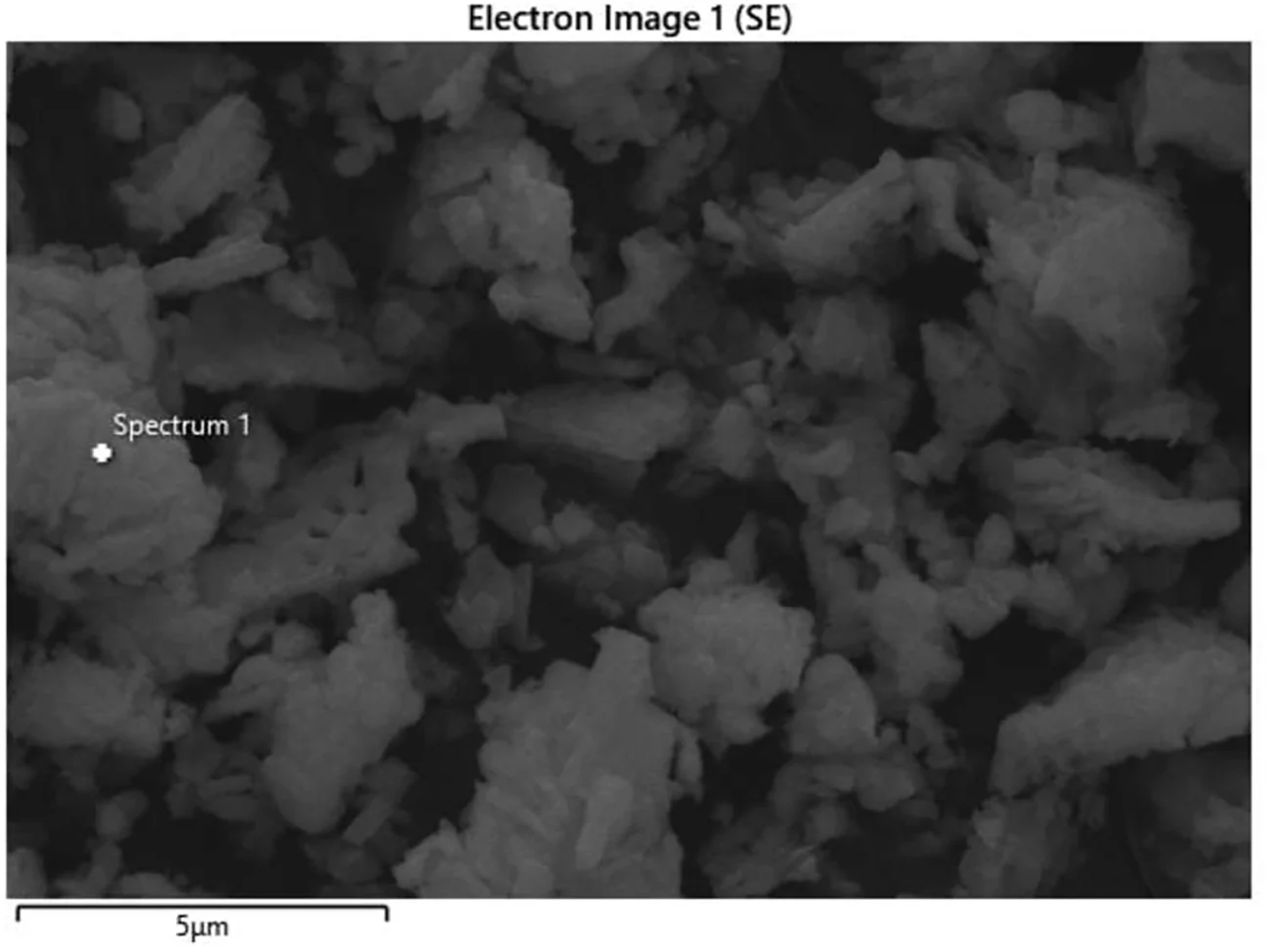
FE-SEM of lanthanum nanoparticles.

The surface morphology of the green-synthesized La_2_O_3_ nanoparticles was examined by FESEM at different magnifications to evaluate particle morphology, size distribution, surface characteristics, and aggregation behavior (**Figure 6**). At lower magnification (5,000×), the sample exhibited a dense and relatively homogeneous distribution of nanoparticle aggregates across the examined area, with no apparent large-scale phase separation or bulk crystalline structures. The particles appeared predominantly aggregated, which may be attributed to the high surface energy of nanoscale particles and the presence of residual phytochemical components originating from the orange peel extract.

At intermediate magnification (25,000×), individual particle domains became more distinguishable and revealed an irregular, flake-like to quasi-spherical morphology. The particles were not uniformly spherical and showed variation in shape and apparent dimensions. Such morphological heterogeneity may arise from differences in nucleation and growth rates during the plant-mediated synthesis and from interactions between La species and phytochemical constituents present in the extract.

At higher magnification (50,000× and100,000×,(Supplementary-1)), finer structural features and individual nanoparticle boundaries were more clearly resolved. Although some primary particles could be distinguished, considerable aggregation was observed, resulting in clusters of closely associated nanoparticles. Importantly, the apparent increase in particle size or aggregation observed at lower magnifications should not be interpreted as a change in the intrinsic particle size; rather, it reflects the visualization of nanoparticle assemblies and agglomerates at different spatial scales.

Overall, the FESEM images demonstrate that the synthesized La_2_O_3_ nanoparticles possessed predominantly irregular, flake-like and quasi-spherical morphology with appreciable aggregation. The variation in morphology observed across magnifications is consistent with a heterogeneous nucleation and growth process mediated by the complex mixture of phytochemicals present in *C. sinensis* peel extract.

### 3.5 Energy-dispersive X-ray spectroscopy

**Figure 7:**
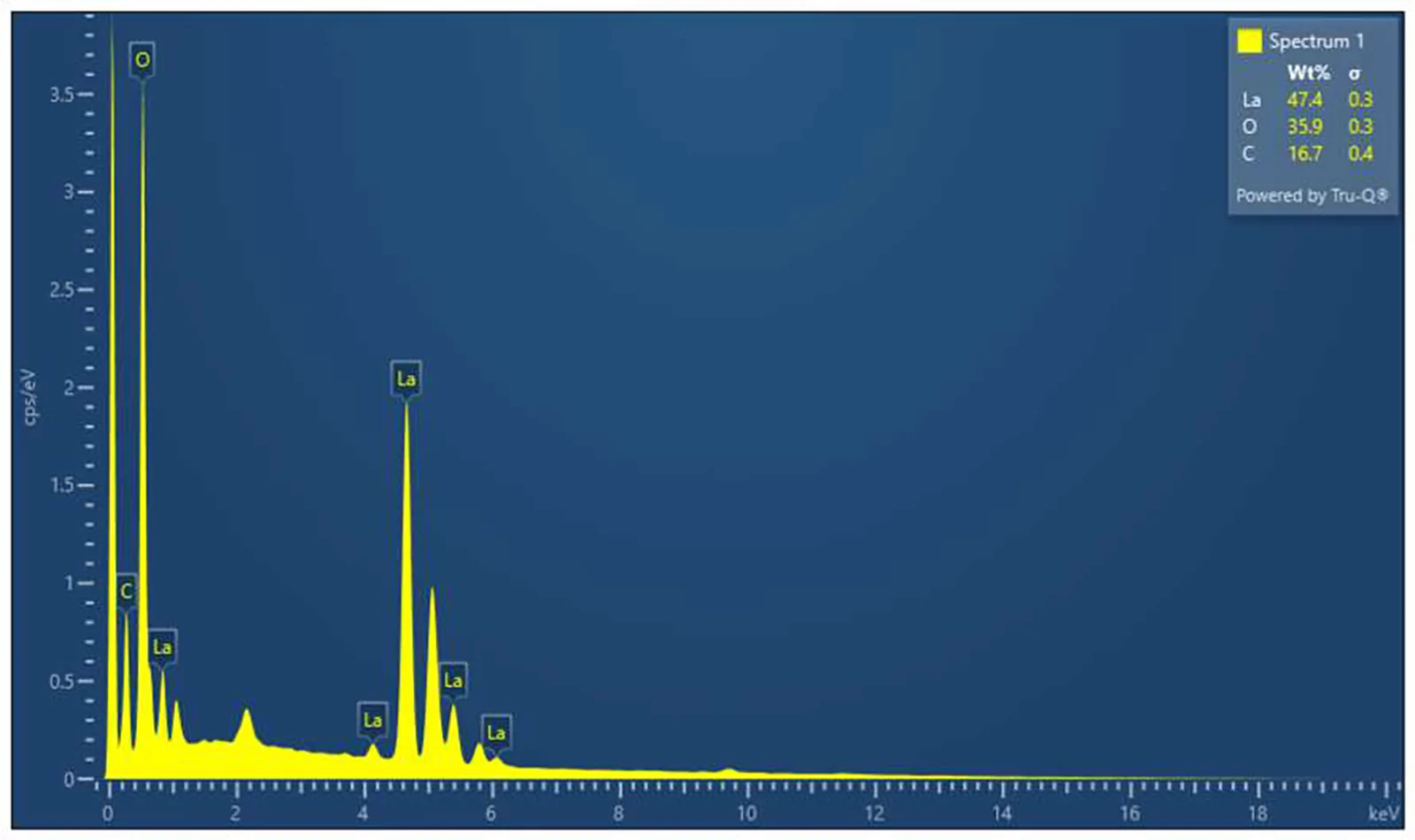
Energy-dispersive X-ray spectroscopy (EDX) of lanthanum oxide nanoparticles.

The elemental composition of the synthesized La_2_O_3_ nanoparticles was determined by EDX spectroscopy (**Figure 7**). The spectrum showed distinct peaks corresponding to oxygen (O), lanthanum (La), and carbon (C), with lanthanum exhibiting characteristic peaks in both the low-energy (~4–5 keV) and higher-energy (~5.5–6.5 keV) regions of the spectrum, consistent with its Lα and Lβ emission lines. Quantitative analysis (Spectrum 1) revealed a composition of 47.4 ± 0.3 wt% lanthanum, 35.9 ± 0.3 wt% oxygen, and 16.7 ± 0.4 wt% carbon. No peaks corresponding to elements other than La, O, and C were detected, indicating the absence of extraneous elemental contamination in the synthesized sample.

### 3.6 Antifungal activity assay

The antifungal efficacy of La_2_O_3_ nanoparticles (NPs) varied substantially between the two evaluated phytopathogens, demonstrating a distinct concentration-dependent response (**Table 1**). One-way ANOVA confirmed that the overall treatment effects were statistically highly significant (*p* < 0.001) for both *Alternaria solani* and *Colletotrichum capsici*.

**Table 1:** Inhibitory effects of La_2_O_3_ nanoparticles and commercial fungicide on the radial mycelial growth of *Alternaria solani* and *Colletotrichum capsici*.

Treatment	Concentration	Mycelial growth inhibition (%) *Alternaria solani*	Mycelial growth inhibition (%) *Colletotrichum capsici*
Untreated Control	—	0.0 ± 0.0	0.0 ± 0.0
La_2_O_3_	1 mg/mL	24.4 ± 3.1	9.9 ± 2.4
La_2_O_3_	5 mg/ml	100.0 ± 0.0	61.5± 3.8
La_2_O_3_	15 mg/ml	100.0 ± 0.0	86.8 ± 2.1
SAAF Fungicide	5 mg/ml	100.0 ± 0.0	100.0 ± 0.0

For *A. solani*, complete suppression of mycelial growth (100.0 ± 0.0%) was achieved at both the (5 mg/mL and 15 mg/mL) nanoparticle concentrations, which completely matched the performance of the commercial SAAF fungicide control (100 ± 0.0%). At the lowest tested concentration of (1mg/mL), the La_2_O_3_ NPs displayed a clear, independent inhibitory effect, restricting mycelial expansion by (24.4 ± 3.1%) relative to the untreated control.

By contrast, *C. capsici* presented a more progressive, incremental susceptibility profile across the treatment gradient. Complete growth restriction (100.0 ± 0.0%) was restricted solely to the commercial SAAF fungicide treatment. However, increasing concentrations of La_2_O_3_ NPs resulted in increased, stepwise inhibition of the mycelium, rising from (61.5 ± 3.8%) at (5 mg/mL) to a substantial suppression level of (86.8 ± 2.1%) at (15 mg/mL). At the minimum application rate of (1 mg/mL), the inhibitory effect was minimal (9.9 ± 2.4%) and did not form a statistically significant deviation from the growth observed in the untreated control (*p* < 0.001).

**Figure 8:**
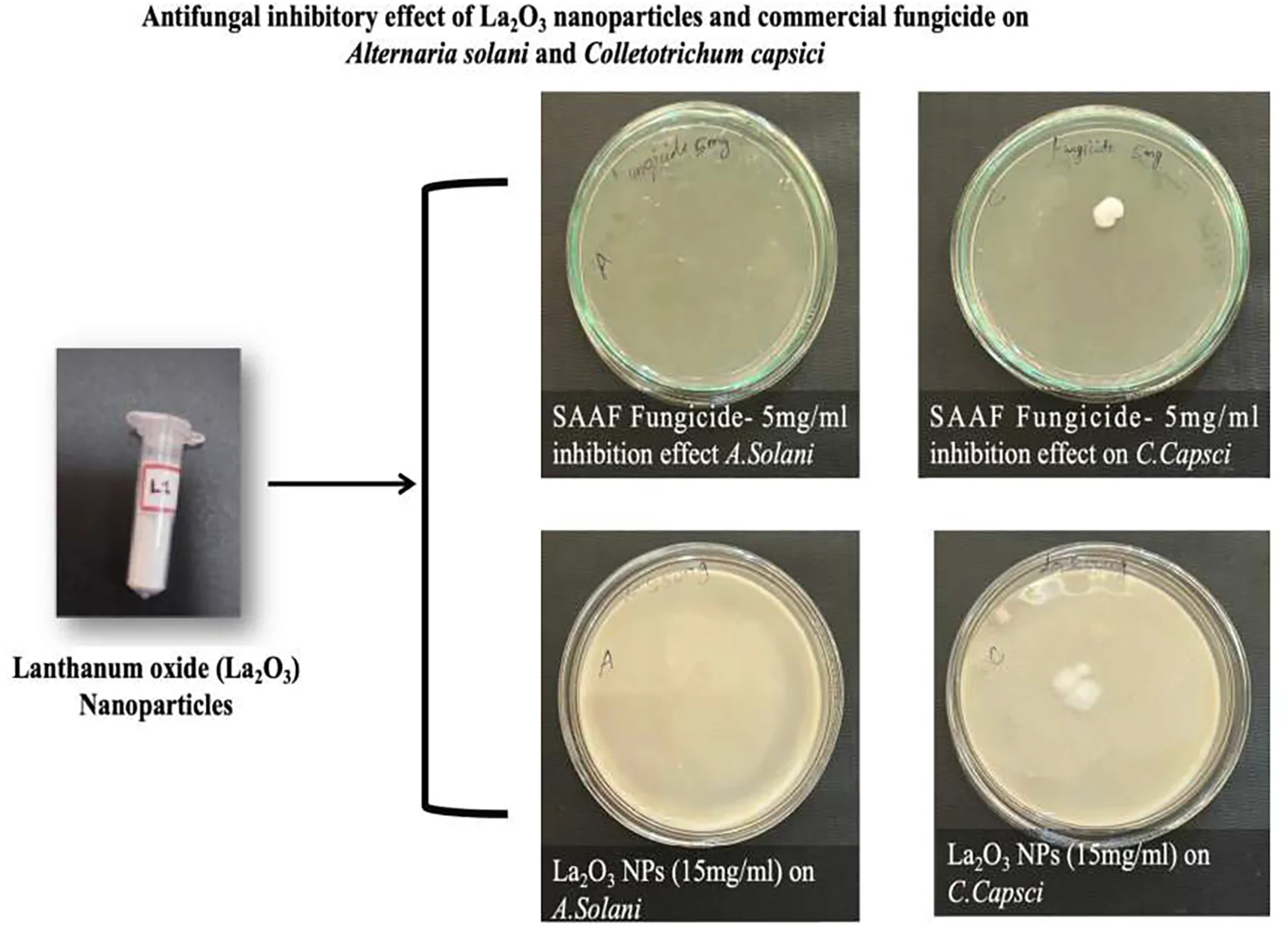
Antifungal inhibitory effect of La_2_O_3_ nanoparticles and commercial fungicide on *Alternaria solani* and *Colletotrichum capsici* at 5 mg/mL (fungicide) and 15 mg/mL (La_2_O_3_).

Each value represents the mean ± standard deviation (SD) of three independent replicates (n = 3). The overall treatment effect for both pathogens was statistically highly significant (One-Way ANOVA, (*p* < 0.001). The antifungal inhibitory effects of La_2_O_3_ nanoparticles and the commercial fungicide against A. solani and C. capsici are presented in **Figure 8**.

## 4 Discussion

The colorimetric changes observed during synthesis provide visual evidence of the underlying chemical transformations involved in nanoparticle formation. The initial color change from dark yellow to pale yellow reflects electron transfer from polyphenolic reductants present in the orange peel extract to lanthanum(III) ions, consistent with the electron-donating capacity of hydroxyl-rich phenolic compounds (Pandey and Rizvi, 2009; Dai and Mumper, 2010). The tan-colored pellet recovered after centrifugation corresponds to precipitation of a lanthanum hydroxide intermediate (Hanif et al., 2024). The subsequent shift to bright yellow after drying indicates partial oxidation of this intermediate even at moderate temperature (Hanif et al., 2024; Rashmi et al., 2022), while the final conversion to a white powder after calcination at 700 °C is characteristic of pure La_2_O_3_ formation (Hanif et al., 2024). Together, these transformations track the expected reaction pathway from metal salt to hydroxide intermediate to crystalline oxide.

The sharp, well-resolved Bragg peaks observed by XRD confirm formation of a highly crystalline, phase-pure cubic La_2_O_3_ (Hanif et al., 2024; Rashmi et al., 2022; Perumal et al., 2025). Perumal et al. (2025), who synthesized La_2_O_3_ using *Ocimum basilicum* extract, reported diffraction peaks at different 2θ positions (15.391°, 27.677°, 39.220°, 48.366°). These positional differences likely arise from variation in crystal orientation, particle size-dependent peak broadening (Hanif et al., 2024), or lattice parameter shifts resulting from differential phytochemical incorporation during synthesis (Mittal et al., 2013; Singh et al., 2016). The consistent achievement of high crystallinity across different citrus and non-citrus plant extracts (Hanif et al., 2024; Rashmi et al., 2022; Perumal et al., 2025) suggests that plant polyphenols broadly support efficient crystalline oxide formation, an advantage of plant-mediated synthesis over some chemical routes that require harsher conditions to achieve comparable crystallinity (Mittal et al., 2013; Singh et al., 2016).

The FTIR data provide complementary evidence for both the composition of the reducing extract and the surface chemistry of the resulting nanoparticles. The orange peel extract spectrum showed bands characteristic of its phytochemical constituents, hydroxyl groups (3740, 3336 cm^–1^), aliphatic C–H (2924 cm^–1^), carbonyl (1735 cm^–1^), aromatic C=C (1604 cm^–1^), and C–O-containing linkages (1245, 1018 cm^–1^), consistent with a polyphenol- and polysaccharide-rich matrix capable of acting as a reducing and capping agent (Pandey and Rizvi, 2009; Dai and Mumper, 2010).

Comparison of the extract and nanoparticle spectra shows that this organic profile is substantially, but not completely, lost during synthesis. The nanoparticle spectrum retained O–H bands at 3740 and 3440 cm^–1^ and showed residual C–O-related bands at 1380 and 1070 cm^–1^, indicating that a fraction of the extract-derived phytochemicals remained bound to the nanoparticle surface even after calcination at 700 °C. This is consistent with the carbon content later confirmed by EDX (Section 4.6) and supports a bio-hybrid nanostructure in which an inorganic oxide core retains a residual organic surface coating (Ahmed et al., 2016; Pandey and Rizvi, 2009). The persistence of the 3740 cm^–1^ band in both spectra is particularly notable, as it suggests this specific hydroxyl environment survives the thermal treatment largely intact.

The emergence of three new low-wavenumber bands in the nanoparticle spectrum, at 890, 620, and 460 cm^–1^, all attributed to La–O stretching vibrations, provides direct spectroscopic confirmation of oxide lattice formation, corroborating the XRD results. Hanif et al. (2024), using *C. aurantium* leaf extract, reported a single La–O band in the 455–516 cm^–1^ range. The 460 cm^–1^ band observed here falls within that reported range, while the two additional bands at 620 and 890 cm^–1^ were not reported in that study. This more resolved La–O vibrational profile may reflect differences in particle size, crystallite morphology, or the specific coordination environment created by *C. sinensis*-derived phytochemicals during synthesis (Hanif et al., 2024; Rashmi et al., 2022).

The UV absorption maximum at approximately 360 nm is consistent with literature values reported for La_2_O_3_ nanoparticles synthesized by other green routes (Chakravorty et al.,2025; Rashmi et al., 2022; Perumal et al., 2025), indicating that the nanoparticles retain the expected electronic structure of the bulk oxide despite their nanoscale dimensions and the ambient-temperature initiation of the synthesis route. The minimal absorbance recorded across the visible region (400–700 nm) is characteristic of wide-bandgap metal oxides and confirms that any photocatalytic response of these nanoparticles would occur predominantly under UV excitation rather than visible light. The absorption shoulder observed at 290–320 nm is most plausibly attributed to residual flavonoid compounds from the orange peel extract, which characteristically absorb in this range due to π→π* and n→π* transitions within their aromatic ring systems (Pandey and Rizvi, 2009; Dai and Mumper, 2010). This shoulder provides further optical evidence, alongside the FTIR data, of retained organic constituents on the nanoparticle surface.

The irregular, flake-like, quasi-spherical morphology observed by FESEM differs from the uniform spherical morphology (mean size 51.41 nm) reported by Hanif et al. (2024) for La_2_O_3_ nanoparticles synthesized using *C. aurantium* leaf extract. This variation in particle shape likely reflects differences in extraction solvent, extract concentration, or synthesis pH between the two studies (Hanif et al., 2024; Rashmi et al., 2022; Mittal et al., 2013; Singh et al., 2016), factors known to influence nucleation and growth kinetics during green synthesis. The tendency of primary particles to aggregate into clusters, while individual particle boundaries remain discernible, is consistent with the high surface-to-volume ratio characteristic of nanoscale particles and is likely promoted by bio-capping agents derived from the orange peel extract, which act as stabilizing interfaces between adjacent particles without fully preventing aggregation (Ahmed et al., 2016; Pandey and Rizvi, 2009).

The EDX-derived composition (47.4 ± 0.3 wt% La, 35.9 ± 0.3 wt% O, 16.7 ± 0.4 wt% C) confirms successful oxide formation while providing direct quantitative evidence for the organic surface coating inferred from the FTIR data (Section 4.3). The substantial carbon content indicates retention of plant-derived secondary metabolites, polyphenols, carbohydrates, and related compounds, that served as reducing and capping agents during synthesis (Ahmed et al., 2016; Pandey and Rizvi, 2009; Dai and Mumper, 2010). This finding, together with the persistence of O-H and C-O bands in the nanoparticle FTIR spectrum, supports a bio-hybrid nanostructure model in which an inorganic La_2_O_3_ core is coated with residual bioactive phytochemicals (Hanif et al., 2024; Ahmed et al., 2016). It is worth noting that the measured La:O weight ratio deviates from the theoretical stoichiometric ratio for pure La_2_O_3_; this is a common observation in EDX analysis of green-synthesized nanoparticles and likely reflects both the inherent limitations of EDX for quantifying light elements and the diluting effect of the retained organic surface layer on the inorganic signal. The absence of extraneous elemental peaks nonetheless indicates that the synthesized nanoparticles are free of contamination from unintended sources (Hanif et al., 2024; Rashmi et al., 2022; Perumal et al., 2025). This retained phytochemical fraction is consistent with reports that *C. sinensis* peel extract alone possesses direct antifungal activity against phytopathogenic fungi (Hajji-Hedfi et al., 2025), suggesting that the surface-bound organic layer identified by FTIR and EDX in this study may itself contribute an independent antifungal component, alongside the oxidative and cell-wall-disruptive mechanisms attributed to the inorganic La_2_O_3_ core. *A.solani* was markedly more sensitive to La_2_O_3_ nanoparticles than *C. capsici*, achieving complete growth inhibition at 5 mg/mL, whereas *C. capsici* reached only 86.8% inhibition even at the highest concentration tested (15 mg/mL). This pathogen-specific difference is most plausibly explained by differences in fungal cell wall architecture (Oliveira Silva et al., 2022). *A.solani*, an ascomycete pathogen, possesses relatively thin, loosely organized cell walls composed primarily of glucans and chitin with minimal melanization (Than et al., 2008; Curto et al., 2021; Antunes et al., 2024), a structure that would permit greater nanoparticle penetration and correspondingly greater exposure to nanoparticle-generated reactive oxygen species (ROS) (Slavin and Bach, 2022).

*Colletotrichum* species, by contrast, possess thicker, more heavily melanized cell walls (Than et al., 2008; Jia et al., 2025) melanin is a recognized ROS scavenger (Slavin and Bach, 2022), which would be expected to reduce the bioavailability of nanoparticle-induced oxidative stress at the cell surface and confer partial resistance (Ramachandra et al., 2023). Despite this, the 86.8% inhibition achieved against *C. capsici* at 15 mg/mL represents a substantial degree of suppression against a comparatively difficult-to-control pathogen (Muhae‐Ud‐Din et al., 2025), and indicates that the antifungal mechanism, most plausibly a combination of oxidative stress generation and direct contact-induced cell wall disruption (Oso et al., 2025). The complete suppression of sporulation observed across all La_2_O_3_-treated plates, for both pathogens (Perumal et al., 2025; Kishore et al., 2025b) is a further indication of antifungal efficacy beyond simple growth restriction, since spore production is central to secondary disease spread in field conditions (Sánchez Espinosa et al., 2024). Against *A. solani*, the nanoparticles at 15 mg/mL achieved complete inhibition two days earlier than the commercial fungicide SAAF at 5 mg/mL (Day 2 vs. Day 4) (Fisher et al., 2018).

While both treatments ultimately achieved 100% inhibition, this kinetic advantage may be practically meaningful, since more rapid early-stage growth arrest during the critical infection window is expected to limit disease severity and associated crop yield loss under field conditions.

Against *C. capsici*, however, the commercial fungicide outperformed the nanoparticles in terms of final efficacy, achieving complete inhibition at 5 mg/mL compared to the 86.8% inhibition obtained with nanoparticles even at 15 mg/mL. This difference suggests that the multi-component fungicide formulation (Carbendazim 12% + Mancozeb 63% WP), which combines a systemic benzimidazole fungicide with a broad-spectrum protectant, provides a mode of action better suited to overcoming the structural defenses of heavily melanized, more resistant pathogens such as *Colletotrichum* species (Fisher et al., 2018).

These findings indicate that green-synthesized La_2_O_3_ nanoparticles perform comparably to, and with faster kinetics than, a commercial fungicide against *A. solani*, while providing substantial though incomplete control of *C. capsici*. These results offer proof-of-concept evidence for La_2_O_3_ nanoparticles as a viable, eco-friendly biocontrol agent, particularly for management of early blight, while indicating that further optimization, such as higher application concentrations, extended exposure times, or combination with complementary antifungal agents, may be required to achieve comparable efficacy against more resistant pathogens like *C. capsici*.

## 5 Conclusion

The present study demonstrated the successful green synthesis of La_2_O_3_ nanoparticles using *Citrus sinensis* peel waste as a sustainable biological source, confirming their crystalline structure and characteristic physicochemical properties. The synthesized nanoparticles exhibited concentration-dependent antifungal activity against *Alternaria solani* and *Colletotrichum capsici*, with *A. solani* showing greater susceptibility and complete growth inhibition at 5 mg/mL, whereas inhibition of *C. capsici* reached 86.8% at 15 mg/mL. Although the activity was lower than that of the commercial fungicide SAAF, the findings indicate that peel-derived La_2_O_3_ nanoparticles have promising potential as an eco-friendly antifungal agent.

## Data Availability

The original contributions presented in the study are included in the article/**Supplementary Material**. Further inquiries can be directed to the corresponding author.
