# Supplementary material for: Biogenic synthesis and characterization of lanthanum oxide nanoparticles using citrus sinensis peel waste for antifungal applications in crop protection
Source: Front Fungal Biol. 2026 Sep 11;7:1925291. doi: 10.3389/ffunb.2026.1925291 (PMC13612435; doi:10.3389/ffunb.2026.1925291)
Supplement: Supplementary file 1 [file Supplementaryfile1.docx]

**Supplementary Material**

**Supplementary Figure S1.** FESEM micrograph of green-synthesized La2O3 nanoparticles at 5,000× magnification

**Supplementary Figure S2.** FESEM micrograph of green-synthesized La2O3 nanoparticles at 6,000× magnification.

**Supplementary Figure S3.** FESEM micrograph of green-synthesized La2O3 nanoparticles at 25,000× magnification.

**Supplementary Figure S4.** FESEM micrograph of green-synthesized La2O3 nanoparticles at 50,000× magnification.

**Supplementary Figure S5.** FESEM micrograph of green-synthesized La2O3 nanoparticles at 50,000× magnification.

Supplementary Figure S6. FESEM micrograph of green-synthesized La2O3 nanoparticles at 100,000× magnification.
